# Revefenacin, a once-daily, long-acting muscarinic antagonist, for nebulized maintenance therapy in patients with chronic obstructive pulmonary disease

**DOI:** 10.1093/ajhp/zxab154

**Published:** 2021-04-04

**Authors:** Christopher Hvisdas

**Affiliations:** Department of Pharmacy, Penn Presbyterian Medical Center, University of Pennsylvania, Philadelphia, PA, USA

**Keywords:** anticholinergics, bronchodilators, chronic obstructive pulmonary disease, drug administration, drug development and approval, revefenacin

## Abstract

**Purpose:**

This article reviews the efficacy and safety of revefenacin, the first once-daily, long-acting muscarinic antagonist, when delivered via a standard jet nebulizer in patients with chronic obstructive pulmonary disease (COPD).

**Summary:**

Revefenacin 175 µg is indicated for the maintenance treatment of patients with moderate to very severe COPD. Preclinical studies showed that revefenacin is a potent and selective antagonist with similar affinity for the different subtypes of muscarinic receptors (M_1_-M_5_). Furthermore, prevention of methacholine- and acetylcholine-induced bronchoconstrictive effects was dose dependent and lasted longer than 24 hours, demonstrating a long duration of action. In phase 2 and 3 trials, treatment with revefenacin was demonstrated to result in statistical improvements in pulmonary function (≥100 mL, *P* < 0.05) vs placebo, including among patients with markers of more severe disease and those who received concomitant long-acting β-agonists or long-acting β-agonists together with inhaled corticosteroids. Revefenacin was also demonstrated to have efficacy similar to that of tiotropium. The clinical trial findings indicated no significant difference between revefenacin and tiotropium with regard to rates of adverse events. Overall, revefenacin was well tolerated, with COPD worsening/exacerbation, dyspnea, headache, and cough among the most common adverse events noted in the clinical trials.

**Conclusions:**

Revefenacin treatment delivered via nebulization led to improvements in lung function in patients with COPD. It was also generally well tolerated, with no major safety concerns. Revefenacin provides a viable treatment option for patients with COPD and may be a suitable alternative for those with conditions that may impair proper use of traditional handheld inhalers.

**Key Points**

- Revefenacin is the only once-daily nebulized long-acting muscarinic antagonist approved by the Food and Drug Administration for the maintenance treatment of chronic obstructive pulmonary disease.
- Compared with placebo, revefenacin demonstrated statistically clinically important improvements in pulmonary function.
- Revefenacin was well tolerated; worsening/exacerbation of chronic obstructive pulmonary disease, cough, dyspnea, and headache were among the most common adverse events noted in clinical trials.

Revefenacin is the first once-daily long-acting muscarinic antagonist (LAMA) for use with a standard jet nebulizer indicated for the maintenance treatment of patients with chronic obstructive pulmonary disease (COPD).^1^ In the United States, approximately 16.4 million adults have a confirmed diagnosis of COPD, and it is the fourth leading cause of mortality, with an estimated annual cost of $49.9 billion.^2-4^

The Global Initiative for Chronic Obstructive Lung Disease (GOLD) report offers guidance on the diagnosis and management of COPD. Inhaled bronchodilators are recommended as first-line therapy for the treatment of COPD.^5^ Although GOLD does not recommend a particular bronchodilator over another, evidence suggests that LAMAs offer clinical and economic benefits, compared with long-acting β-agonists (LABAs). Long-acting inhaled bronchodilators are most often administered with pressurized metered-dose inhalers or dry powder inhalers (DPIs). However, patients with cognitive or physical limitations or with suboptimal peak inspiratory flow rate (PIFR) may have challenges with inhalers.^6,7^ These patients may benefit from nebulized therapy, which may improve symptom control, compared with other delivery devices.^8^ Until recently, there was only 1 nebulized LAMA available for twice-daily administration, glycopyrrolate bromide (Lonhala Magnair, Pari, Munich, Germany).^9^

The Food and Drug Administration (FDA) approved the use of revefenacin (Yupelri, Theravance Biopharma, South San Francisco, CA) in November 2018.^1^ Clinical trial data for revefenacin were obtained using the Pari LC Sprint nebulizer (Pari, Starnberg, Germany) and the Pari Trek S compressor (Pari, Midlothian, VA). The pharmacology, pharmacokinetics (PK), efficacy, safety, and clinical application of revefenacin are reviewed in this article, with a focus on the FDA-approved 175-µg dose. Information on the data selection, revefenacin dosage and administration, and revefenacin drug interactions is provided in the eAppendix.

## Pharmacology and PK profile

 Revefenacin is a nonester, nonquaternary ammonium–based LAMA. The terminal amide in revefenacin’s structure provides a metabolically labile functionality, which appears to be stable in the lung but readily hydrolyzed to its active metabolite in systemic circulation,^10^ thus potentially minimizing systemically mediated adverse events (AEs).

Similar to tiotropium,^11^ revefenacin is a potent and selective antagonist, with similar affinity to the subtypes of muscarinic receptors (M_1_-M_5_).^12^ Revefenacin exhibits pharmacological effects through the inhibition of the M_3_ receptor at the airway smooth muscle, thereby leading to bronchodilation.^12^ M_3_ receptors are found on bronchial smooth muscle and mediate bronchoconstriction; thus, in theory, M_3_ antagonism results in bronchodilation. In preclinical studies, prevention of methacholine- and acetylcholine-induced bronchoconstrictive effects was dose dependent and lasted more than 24 hours,^13^ demonstrating a long duration of action.

After inhaled administration of revefenacin in patients with COPD, conversion to the metabolite THRX-195518 occurred rapidly, and plasma exposures of THRX-195518 were approximately 3- to 6-fold greater than those for revefenacin.^14^ THRX-195518 is produced by hepatic metabolism and has lower activity (approximately one third to one tenth) at target muscarinic receptors than revefenacin.^1,14^

## Dosing in renal, hepatic, and cardiac disease

The effects of severe renal impairment (estimated glomerular filtration rate <30 mL/min/1.73 m^2^) and moderate hepatic impairment (Child-Pugh class B) on revefenacin PK were assessed in study volunteers in 2 multicenter, open-label, parallel-group, phase 1 trials (ClinicalTrials.gov identifiers, NCT02581592 and NCT02578082).^15^ Study volunteers received a single 175-µg dose of revefenacin via nebulization.

Systemic exposure to revefenacin was modestly increased by severe renal impairment, while exposure to THRX-195518 was approximately 2-fold higher than in healthy volunteers. In individuals with moderate hepatic impairment, systemic exposure to revefenacin was similar to that in individuals with normal hepatic function, while exposure to THRX-195518 was approximately 3-fold higher. The increase in systemic exposure to THRX-195518 in individuals with severe renal or moderate hepatic impairment was considered unlikely to be of clinical consequence given its low antimuscarinic potency, low systemic levels after inhaled revefenacin, and favorable safety profile.^15^

Cardiac safety was assessed in healthy volunteers in a randomized, 4-way crossover phase 1 trial (NCT02820311).^16^ Each healthy volunteer received a single dose of the following 4 treatments in separate treatment periods: blinded revefenacin 175 µg, revefenacin 700 µg, placebo via nebulization, and open-label oral moxifloxacin 400 mg (positive control). Revefenacin did not have a clinically meaningful effect on cardiac repolarization or cardiac conduction and was generally well tolerated.^16^

## Clinical trials

The methodology and results of 4 phase 2 studies and 5 phase 3 studies are summarized and discussed in Table 1 and Table 2.^14,17-23^ The results of 2 post hoc/prespecified studies are summarized and discussed in Table 3.^24,25^ Eligibility criteria and definitions for phase 2 and 3 clinical trials are discussed in the eAppendix.

**Table 1.** Summary of Phase 2 and Phase 3 Clinical Trials^a^

Clinical Trial	Study Design	Intervention	Duration	Baseline Characteristics
Study 0091 (NCT01704404)^14^	Phase 2 Randomized, double-blind, placebo-controlled, multiple-dose, incomplete block, 5-way crossover design *n* = 59	REV 22, 44, 88, 175, 350, or 700 μg or PBO OD	7 days	Men: 56% Mean age: 64 years Mean FEV_1_ (percentage of predicted normal): 47%
Study 0116 (NCT02109172)^17^	Phase 2 Randomized, double-blind, placebo-controlled, dose-ranging, crossover design *n* = 64	REV 44 BID or 175 μg OD or PBO OD or BID	7 days	Information not available online
Study 0117 (NCT02040792)^18^	Phase 2 Randomized, double-blind, placebo-controlled, dose-ranging design *n* = 355	REV 44, 88, 175, or 350 μg or PBO OD	28 days	Men: 50% Mean age: 62 years Mean FEV_1_ (percentage of predicted normal): 44%
Study 0126 (NCT02459080)^19^	Phase 3 Randomized, double-blind, placebo-controlled, multiple-dose, parallel-group design *n* = 619	REV 88 or 175 μg or PBO OD	12 weeks	Men: 47%-52% Mean age: 64 years Mean FEV_1_ (percentage of predicted normal): 54-56% Current smoker: 48%-49%
Study 0127 (NCT02512510)^19^	Phase 3 Randomized, double blind, placebo-controlled, multiple-dose, parallel-group design *n* = 611	REV 88 or 175 μg or PBO OD	12 weeks	Men: 47%-52% Mean age: 63-64 years Mean FEV_1_ (percentage of predicted normal): 54% Current smoker: 45%-48%
Study 0128 (NCT02518139)^20,21^	Phase 3 Randomized, partially double-blinded, parallel-group design *n* = 1,020	REV 88 or 175 μg or TIO 18 μg via HandiHaler OD	52 weeks	Men: 56%-61% Mean age: 64-65 years Mean FEV_1_ (percentage of predicted normal): 53-54% Current smoker: 45%-47%
Study 0149 (NCT03095456)^22^	Phase 3b Randomized, double-blind, active comparator, parallel-group design *n* = 206	REV 88 or 175 μg or TIO 18 μg via HandiHaler OD	28 days	Men: 60% Mean age: 65 years Mean FEV_1_ (percentage of predicted normal): 37% Current smoker: 47%
Study 0167 (NCT03573817)^23^	Phase 3b Randomized, double-blind, 2-period, parallel-group design *n* = 122	REV 175 μg OD, FOR 20 μg BID Sequential administration for 21 days (days 1-21): REV administered in the morning followed by FOR in the morning; FOR alone administered in the evening Combined administration for 21 days (days 22-42): REV and FOR administered together in the morning as a mixed solution; FOR administered alone in the evening	42 days	Men: 56%-58% Mean age: 63-64 years Mean FEV_1_ (percentage of predicted normal): 55% Current smoker: 54%-59%

Abbreviations: BID, twice daily; FEV_1_, forced expiratory volume in 1 second; FOR, formoterol; OD, once daily; PBO, placebo; REV, revefenacin; TIO, tiotropium.

^a^See the eAppendix for information on eligibility criteria, definitions, and criteria for clinical relevance.

**Table 2.** Summary Data From Phase 2 and Phase 3 Clinical Trials of Revefenacin 175 µg for Moderate to Very Severe COPD^a^

Phase	Trial	Key Efficacy Outcomes	Safety Outcomes
Phase 2	Study 0091 (NCT01704404)^14^	Significant improvement in trough FEV_1_ at day 7 (114.2 mL, *P* < 0.001)	Frequency of AEs was lower for REV (45.9%) vs PBO (54.1%) Most common AEs: • Headache (REV, 10.8%; PBO, 14.8%) • Cough (REV, 5.4%; PBO, 1.6%) • Dyspnea (REV, 5.4%; PBO, 6.6%) No antimuscarinic AEs were observed
	Study 0116 (NCT02109172)^17^	Improvement in weighted mean (0-24 hours) FEV_1_ at day 7 (113 mL)	Not assessed
	Study 0117 (NCT02040792)^18^	Significant improvement in trough FEV_1_ at day 28 (166.6 mL, *P* < 0.001)	Frequency of AEs was the same for REV (31.0%) and PBO (31.0%) Most common AEs: • Headache (REV, 1.4%; PBO, 2.8%) • Dyspnea (REV, 4.2%; PBO, 2.8%) • Cough (REV, 4.2%; PBO, 1.4%) No antimuscarinic AEs were observed
Phase 3	Study 0126 (NCT02459080)^19^ Study 0127 (NCT02512510)^19^	Significant improvements in trough FEV_1_ at day 85: • Study 0126: 146.3 mL, *P* < 0.0001 • Study 0127: 147.0 mL, *P* < 0.0001 Significant improvement in peak FEV_1_ at day 85 (pooled studies 0126 and 0127: 129.5 mL, *P* < 0.0001)	Frequency of AEs was similar for REV (51.0%) and PBO (51.7%) Most common AEs: • Worsening/exacerbation of COPD (REV, 10.6%; PBO, 11.0%) • Dyspnea (REV, 2.0%; PBO, 5.3%) • Headache (REV, 4.0%; PBO, 2.4%) • Cough (REV, 3.5%; PBO, 3.8%) Antimuscarinic AEs (constipation and dry mouth) occurred in ≤1% of patients who received REV
	Study 0128 (NCT02518139)^20,21^	Sustained significant improvements from baseline in trough FEV_1_ over 52 weeks for REV (52.3-124.3 mL, *P* < 0.0003) and TIO (79.7-112.8 mL, *P* < 0.0003) Sustained significant improvements (*P* < 0.05) in SGRQ, CAT, CCQ, BDI, and TDI from 3 months on for REV and TIO	Frequency of AEs was lower with REV (72.2%) vs TIO (77.2%) Most common AEs: • Worsening/exacerbation of COPD (REV, 21.8%; TIO, 28.1%) • Nasopharyngitis (REV, 7.8%; TIO, 4.8%) • Upper respiratory tract infection (REV, 6.0%; TIO, 6.8%) • Cough (REV, 7.5%; TIO, 5.6%) Antimuscarinic AEs were lower with REV (2.1%) vs TIO (4.2%): • Dry mouth (REV, ≤0.9%; TIO, 2.8%) • Constipation (REV, 0.9%; TIO, 2.0%)
	Study 0149 (NCT03095456)^22^	Significant improvement in trough FEV_1_ from baseline for REV vs TIO (17.0 mL, *P* = 0.4461) at day 29 Improvement in trough FVC from baseline for REV vs TIO (71.5 mL) at day 29 For patients with predicted FEV_1_ <50%, improvement in trough FEV_1_ for REV vs TIO (49.1 mL) at day 29 For patients with predicted FEV_1_ <50%, improvement in trough FVC for REV vs TIO (103.5 mL) at day 29	Frequency of AEs was lower with REV (11.1%) vs TIO (37.5%) Antimuscarinic AEs were lower in patients who received REV vs TIO: • Constipation (REV, 0%; TIO, 3.8%) • Dry mouth (REV, 1.9%; TIO, 1.0%) Discontinuations resulting from AEs were only reported for TIO (4.8%)
	Study 0167 (NCT03573817)^23^	Improvements from baseline in trough FEV_1_ for REV/FOR during sequential (157.1 mL) and combined (115.6 mL) administration at days 21 and 42, respectively	Frequency of AEs was lower with REV/FOR (sequential, 4.8%; combined, 8.1%) vs PBO/FOR (sequential, 11.9%; combined, 10.9%) The most common AEs (≥2%) occurred in the PBO/FOR groups: • Cough (3.6%) • Worsening of COPD (3.4%) • Dizziness (3.4%) AEs that led to discontinuation for REV/FOR occurred in ≤1.6% of patients

Abbreviations: AE, adverse event; BDI, Baseline Dyspnea Index; CAT, COPD Assessment Test; CCQ, Clinical COPD Questionnaire; COPD, chronic obstructive pulmonary disease; FEV_1_, forced expiratory volume in 1 second; FOR, formoterol; FVC, forced vital capacity; PBO, placebo; REV, revefenacin; SGRQ, St. George’s Respiratory Questionnaire; TDI, Transition Dyspnea Index; TIO, tiotropium.

^a^See the eAppendix for information on eligibility criteria, definitions, and criteria for clinical relevance.

**Table 3.** Summary of Phase 3 Post Hoc/Prespecified Subgroup Analyses^a^

Study Design	Patient Population/ Treatments	Efficacy/Health Status Outcomes	Safety Outcomes
Post hoc subgroup efficacy analysis of phase 3 studies 0126 and 0127^24^	REV 175 µg (*n* = 395) PBO (*n* = 417) The following subgroups of patients with severe markers of COPD were analyzed: • Severe airflow limitation (percent predicted FEV_1_ of 30% to <50%) • Very severe airflow limitation (percent predicted FEV_1_ <30%) • 2011 GOLD D • Patients who were reversible (≥12% in percent predicted FEV_1_) to SABAs (ipratropium and albuterol) • Background ICS • Background LABA and/or ICS • Older age: ◦ >65 years ◦ >75 years • History of comorbidity risk factors: ◦ Cardiovascular disease ◦ Diabetes mellitus o Cognitive/mental impairments	Clinically significant improvements in day 85 trough FEV_1_ (mL) for REV vs PBO across all subgroups: • Severe airflow limitation (131.2, *P* < 0.001) • Very severe airflow limitation (176.2, *P* = 0.0324) • 2011 GOLD D (124.6, *P* < 0.0001) • Reversibility to SABAs (286.5, *P* < 0.0001) • Background ICS (130.6, *P* < 0.001) • Background LABA and/or ICS (139.2, *P* < 0.0001) • >65 years (140.3, *P* < 0.0001) • >75 years (129.2, *P* = 0.0217) • History of cardiovascular disease (140.7*, P* = 0.0242) • History of diabetes mellitus (101.6, *P* = 0.0077) • History of cognitive/mental impairments (149.5, *P* = 0.0006) For the SGRQ responders, the odds of response (odds ratio >2.0) were significantly greater (and of clinical importance) in the REV arm vs the PBO arm among the following subgroups: • Percent predicted FEV_1_ of 30% to <50% • Percent predicted FEV_1_ <30% • 2011 GOLD D For the TDI responders, the odds of response (odds ratio >2.0) were significantly greater (and of clinical importance) in the REV arm vs the PBO arm among the following subgroups: • Percent predicted FEV_1_ <30% • > 75 years	Not assessed
Prespecified subgroup efficacy analysis (studies 0126 and 0127) and safety analysis (studies 0126, 0127, and 0128)^25^	0126/0127 REV or PBO and concomitant LABA or LABA/ICS REV 175 µg (*n* = 153), PBO (*n* = 147) REV or PBO only REV 175 µg (*n* = 242), PBO (*n* = 270) 0128 REV or TIO and concomitant LABA or LABA/ICS REV 175 µg (*n* = 158), TIO 18 µg (*n* = 177) REV or TIO only REV 175 µg (*n* = 161), TIO 18 µg (*n* = 174)	REV led to similar improvements from baseline in trough FEV_1_ across subgroups Trough FEV_1_ (LS mean difference) at day 85: • REV only: 150.9 mL, *P* < 0.0001 • REV and concomitant LABA or LABA/ICS: 139.2 mL, *P* < 0.0001 Similar improvements were observed in SGRQ scores between subgroups: • REV only: –3.3 • REV and concomitant LABA or LABA/ICS: –3.4	Incidence of AEs • REV only: 37.5% • REV and concomitant LABA or LABA/ICS: 50.2% Exacerbation of COPD was the most commonly reported AE: • Incidence was higher in the subgroup with REV and concomitant LABA or LABA/ICS (25.0%) vs the REV only subgroup (11.8%) Antimuscarinic-related AEs were reported more frequently in the subgroup with concomitant LABA or LABA/ICS (2.5%) vs the REV only subgroup (1.4%) Dry mouth: • REV and concomitant LABA or LABA/ICS: 1.1% • REV only: 1.0% Constipation: • REV and concomitant LABA or LABA/ICS: 1.2% • REV only: 0.6%

Abbreviations: AE, adverse event; COPD, chronic obstructive pulmonary disease; FEV_1_, forced expiratory volume in 1 second; GOLD, Global Initiative for Chronic Obstructive Lung Disease; ICS, inhaled corticosteroid; LABA, long-acting β-agonist; LS, least squares;

PBO, placebo; REV, revefenacin; SABA, short-acting β-agonist; SGRQ, St. George’s Respiratory Questionnaire; TDI, Transition Dyspnea Index; TIO, tiotropium.

^a^See the eAppendix for information on eligibility criteria, definitions, and criteria for clinical relevance.

### Phase 2 studies

In 2 randomized, double-blind, placebo-controlled phase 2 trials (studies 0059 [NCT03064113] and 0091 [NCT01704404]), researchers evaluated the pharmacodynamics, PK, and safety of single-dose (350 and 700 µg) and multiple-dose (22, 44, 88, 175, 350, or 700 μg) revefenacin in patients with moderate to severe COPD.^14^ The FDA-approved 175-µg dose is discussed. In study 0091, patients were randomized to receive once-daily revefenacin (22, 44, 88, 175, 350, or 700 µg) or placebo for 7 days in a double-blind, incomplete block, 5-way crossover design. The primary efficacy endpoint was trough forced expiratory volume in 1 second (FEV_1_) after the final dose (day 7). At baseline, 56% of patients were men, the mean age was 64 years, and the mean percentage predicted FEV_1_ was 47%.^14^

The mean trough FEV_1_ on day 7 was significantly higher for patients receiving revefenacin vs placebo, with a difference of 114 mL (*P* < 0.001) for the FDA-approved dose of 175 μg. Revefenacin demonstrated a long-lasting (≥24 hours) bronchodilator effect and was rapidly absorbed and extensively metabolized, with minimal accumulation after repeated dosing. Revefenacin was well tolerated, and AEs were generally mild. The most common AEs were dyspnea, headache, and cough.^14^

Researchers evaluated the efficacy and safety of revefenacin in 2 dose-ranging phase 2b studies among patients with moderate to severe COPD.^17,18^ Study 0116 (NCT02109172) was a randomized, double-blind, placebo-controlled, 7-day trial that evaluated once-daily (175 µg) and twice-daily (44 µg) revefenacin.^16^ The primary endpoint was change from baseline in weighted mean FEV_1_ during 0 to 24 hours on day 7. Compared with placebo, revefenacin produced clinically significant improvements from baseline in day 7 weighted mean FEV_1_, with a difference of 113 mL for the FDA-approved 175-μg dose.^17^

Study 0117 (NCT02040792) was a randomized, double-blind, placebo-controlled, parallel-group, dose-ranging (44-350 µg), 28-day trial.^18^ The primary endpoint was change from baseline in day 28 trough FEV_1_; inhaled corticosteroids (ICS) and short-acting bronchodilators were permitted. At baseline, 50% of patients were men, the mean age was 62 years, and the mean percentage predicted FEV_1_ was 44%.

Revefenacin 175 µg clinically and significantly improved day 28 trough FEV_1_ vs placebo, with a difference of 166.6 mL. On day 28, the 24-hour weighted mean difference from placebo for FEV_1_ was numerically similar to the respective trough FEV_1_ value, indicating that bronchodilation was sustained for 24 hours after the dose. Furthermore, revefenacin 175 µg decreased albuterol rescue medication usage, by at least 1 albuterol puff per day.^18^

### Phase 3 studies

 Based on the phase 2 data, the pivotal phase 3 studies in patients with moderate to very severe COPD evaluated the efficacy and safety of revefenacin 88 and 175 µg once daily. The FDA-approved 175-µg dose is discussed.

Studies 0126 (NCT02459080) and 0127 (NCT02512510) were randomized, double-blind, placebo-controlled, parallel-group, 12-week studies.^19^ The primary efficacy endpoint was change from baseline in trough FEV_1_ on day 85. Secondary efficacy endpoints included overall treatment effect on trough FEV_1_ and peak FEV_1_ (0-2 hours after the first dose) on day 1. Concomitant LABA-containing therapy (with or without ICS) was permitted in up to 40% of the study population to ensure robust assessments of concurrent therapies used by the participants. Stable doses of ICS without concomitant LABAs were permitted, but LAMAs and short-acting muscarinic antagonists were prohibited. At baseline, 47% to 52% of patients were men, nearly half were current smokers (46%-49%), the mean age was 63 to 64 years, and the mean baseline postbronchodilator percent predicted FEV_1_ was 54% to 56%.^19^

Compared with placebo, revefenacin resulted in clinically significant improvements in trough FEV_1_ at every time point evaluated. The least squares (LS) mean increase in trough FEV_1_ was 146.3 mL (study 0126) and 147.0 mL (study 0127) for revefenacin (*P* < 0.0001) at day 85. Revefenacin increased overall treatment effect on trough FEV_1_ by at least 100 mL vs placebo in both studies. Analysis of pooled results from the 0126 and 0127 studies showed increases in overall treatment effect on FEV_1_ of 142.3 mL for revefenacin. A significant increase in FEV_1_ occurred within 2 hours of the first treatment with revefenacin in both studies (129.5 mL, *P* < 0.0001).^19^

With respect to safety, the overall incidence of AEs was similar for revefenacin (51.0%, 51.8%) and placebo (51.7%, 46.9%) in studies 0126 and 0127, respectively. COPD worsening/exacerbation (≤12.2%), headache (≤6.8%), respiratory infection (≤6.6%), dyspnea (≤5.7%), and cough (≤5.1%) were the most common AEs, with similar frequencies between treatment groups. Antimuscarinic-related AEs were infrequent and occurred at similar rates for the treatment groups in both studies; none of the patients in either study had more than 1 antimuscarinic AE. The most common antimuscarinic AEs were constipation and dry mouth. Although the incidence of serious AEs (SAEs) was similar for revefenacin and placebo in study 0126 (≤6.7%) and study 0127 (≤3.3%), only 2 serious AEs were considered to be related to treatment with revefenacin (0126, 1 SAE of worsening/exacerbation of COPD; 0127, 1 SAE of pneumonia).^19^ In terms of cardiovascular AEs, the incidence of prolonged QT interval was low (pooled 0126 and 0127: revefenacin, 5.9%; placebo, 5.3%). One major adverse cardiovascular event (MACE) was identified for revefenacin (myocardial infarction/unstable angina); however, this was not deemed related to treatment.^26^ While the length of these replicate studies (approximately 12 weeks) does not allow for conclusions on long-term treatment, results from study 0128 help elucidate the long-term safety profile. Key strengths of the studies include the double-blinded design and the similar results in the replicate studies for both primary and secondary endpoints, therefore adding consistency and validity to their outcomes. Furthermore, compared with placebo, revefenacin in the pooled analysis increased trough FEV_1_ by more than 100 mL, which suggests a minimal clinically important difference for FEV_1_.^19^

A post hoc subgroup study was conducted using data from the phase 3 studies 0126 and 0127 (Table 3).^24^ Revefenacin use was associated with significant improvements from baseline in trough FEV_1_ vs placebo (≥100 mL, *P* < 0.05) among patients with markers of more severe COPD. Markers of more severe COPD included severe and very severe airflow limitation (percent predicted FEV_1_ of 30% to <50% and <30%, respectively), 2011 GOLD D classification, reversibility (≥12% and ≥200 mL increase in FEV_1_) to short-acting bronchodilators, concurrent use of LABAs and/or ICS, older age (>65 and >75 years), and comorbidity risk factors (history of cardiovascular disease, diabetes mellitus, and cognitive/mental impairments). There was a greater number of St. George’s Respiratory Questionnaire (SGRQ) and Transition Dyspnea Index (TDI) responders in the majority of the patient subgroups who received revefenacin vs placebo. For the SGRQ responders, the odds of response (odds ratio >2.0) were significantly greater for patients receiving revefenacin vs placebo among subgroups with severe airflow obstruction, very severe airflow obstruction, and 2011 GOLD D classification. For the TDI responders, the odds of response (odds ratio >2.0) were significantly greater among the severe airflow obstruction subgroup and patients more than 75 years of age.^24^

A prespecified subgroup analysis was conducted using data from the phase 3 studies 0126 and 0127 (Table 3).^25^ Patients receiving concomitant revefenacin and LABA or LABA/ICS vs those receiving revefenacin only were evaluated. Revefenacin produced clinically significant improvements from baseline in trough FEV_1_, and these improvements were similar in patients who received LABA or LABA/ICS and those who received concomitant revefenacin only (day 85 trough FEV_1_, 150.9 and 139.2 mL, respectively; *P* < 0.0001). Similar improvements in SGRQ scores were observed among patients who received revefenacin only and those who received concomitant LABA or LABA/ICS (–3.3 and –3.4, respectively).^25^

Study 0128 (NCT02518139) was a randomized, parallel-group, 52-week phase 3 safety trial that compared revefenacin (88 and 175 µg) administered in a double-blind manner and open-label tiotropium 18 µg administered via a HandiHaler (Spiriva HandiHaler, Boehringer Ingelheim, Ridgefield, CT) in patients with moderate to very severe COPD.^20,21^ The FDA-approved 175-µg dose is discussed. Patients who had been using a stable dose of a LABA or LABA/ICS for at least 30 days at screening were permitted to continue that treatment during the study. Patients who were required to initiate a LABA-containing product to treat a COPD exacerbation during the study were permitted to continue that treatment for the remainder of the trial. At baseline, most patients were men (56%-61%), 45% to 47% were current smokers, the mean age was 64 to 65 years, and the mean baseline postbronchodilator percent predicted FEV_1_ was 53% to 54%.^20,21^

The primary endpoint was the safety and tolerability of revefenacin. The incidence of AEs and SAEs was similar among patients treated with revefenacin (AEs, 72.2%; SAEs, 12.8%) and those treated with tiotropium (AEs, 77.2%; SAEs, 16.3%). COPD exacerbation/worsening was the most frequent AE and occurred at a lower proportion for revefenacin vs tiotropium. Although the rate of antimuscarinic-related AEs was low in the treatment groups, these events were slightly less frequent in patients who received revefenacin (2.1%) than in those who received tiotropium (4.2%).^20^ In terms of cardiovascular AEs, the incidence of prolonged QT interval was low with revefenacin (7.7%) and tiotropium (7.3%). Only 1 MACE was considered to be possibly/probably related to revefenacin (atrial fibrillation).^26^ AEs that led to permanent discontinuation were more frequent for patients who received revefenacin (12.2 %) than for those who received tiotropium (9.3%); however, no emergent AE pattern was identified between treatment groups for the patients discontinuing.^20^ A similar percentage of patients who received revefenacin or tiotropium (<2.5%) discontinued treatment due to COPD exacerbation, whereas the percentage of patients who discontinued treatment due to dyspnea was higher with use of revefenacin (1.8%) than with use of tiotropium (0.6%).^20^

Efficacy and health status outcomes were also assessed as exploratory outcomes in study 0128.^21^ These exploratory endpoints included the change in trough FEV_1_ and changes in health outcomes evaluated using general and COPD-specific respiratory symptom rating instruments (SGRQ, COPD Assessment Test [CAT], Clinical COPD Questionnaire [CCQ], Baseline Dyspnea Index [BDI], and Transition Dyspnea Index [TDI), all assessed over 52 weeks.

During the 52-week treatment period, revefenacin and tiotropium elicited sustained significant (all *P* < 0.0003) improvements from baseline in trough FEV_1_. The trough FEV_1_ profile for revefenacin ranged from 52.3 to 124.3 mL, and that for tiotropium ranged from 79.7 to 112.8 mL. There were statistically significant (*P* < 0.05) improvements in all measured health status outcomes from 3 months on (3, 6, 9, and 12 months) vs baseline, in both treatment arms.^21^ Analysis of minimal clinically important difference in response based on SGRQ total score at day 365 revealed a similar percentage of responders to tiotropium (53%) and revefenacin (42%). The percentage of CAT responders were similar in the treatment groups (revefenacin, 48%; tiotropium, 47%). Clinically relevant improvements in SGRQ and TDI scores were demonstrated with use of either revefenacin or tiotropium. However, changes in CAT and CCQ scores did not reach the predetermined thresholds for clinical significance in any group at any time point.^21^ Study limitations included the open-label design for the tiotropium group. Additionally, the ability to draw conclusions on the efficacy of revefenacin vs tiotropium was limited because the study was not designed or powered to demonstrate statistically significant differences between the treatment groups. Larger studies powered to assess efficacy are needed to assess the comparative effects of these 2 treatments.^21^ The strengths of the study included the length of the study (52 weeks), which demonstrated that long-term revefenacin therapy was well tolerated over long periods of time.

A prespecified subgroup study was also conducted using pooled data from the phase 3 studies (0126, 0127, and 0128) to assess the safety of concomitant revefenacin and LABA or LABA/ICS (Table 3).^25^ Revefenacin was well tolerated, with more AEs reported among patients who received concomitant LABA or LABA/ICS than in those who received revefenacin only. COPD exacerbation was the most commonly reported AE, and its incidence was higher in patients who received concomitant revefenacin and LABA or LABA/ICS (25.0%) than in those who received revefenacin only.^25^

Study 0149 (NCT03095456) was a randomized, double-blind, 28-day phase 3b trial that evaluated the efficacy of revefenacin 175 µg vs tiotropium 18 µg administered via a HandiHaler in patients with moderate to very severe COPD and suboptimal PIFR (<60 L/min).^22^ This study was conducted to help clinicians identify a potentially significant subset of patients with COPD, using an inhalation flow rate test to determine whether revefenacin via nebulization could provide increased benefit vs tiotropium via DPI. The primary endpoint was change from baseline in trough FEV_1_ at day 29. A prespecified subgroup analysis was planned to compare efficacy based on airflow obstruction severity in patients with severe to very severe disease. Key secondary efficacy endpoints were the effect of revefenacin vs tiotropium on trough forced vital capacity (FVC) and inspiratory capacity at day 29 and peak FEV_1_ and FVC at day 29 (0-4 hours). Patients were permitted to continue concurrent LABA or LABA/ICS therapy. At baseline, most patients were men (60%), 47% of patients were current smokers, the mean age of the patients was 65 years, and the mean baseline postbronchodilator percent predicted FEV_1_ was 37%.^22^

Revefenacin and tiotropium improved trough FEV_1_ and FVC from baseline on day 29, with better improvements among those receiving revefenacin vs tiotropium; however, the difference in FEV_1_ was not significant (LS mean difference: FEV_1_, 17.0 mL [*P* = 0.4461]; FVC, 71.5 mL). In patients with severe to very severe airflow limitation (predicted FEV_1_ <50%), revefenacin and tiotropium improved trough FEV_1_ from baseline on day 29, with greater improvements among those receiving revefenacin vs tiotropium (LS mean difference: FEV_1_, 49.1 mL; FVC, 103.5 mL). Overall, the differences between the treatments were not clinically meaningful.^22^

Safety was assessed through AE evaluation. A limitation of this trial was its length. Because this was a 4-week trial designed to evaluate the efficacy of revefenacin via nebulization vs tiotropium via DPI, the long-term safety of revefenacin in patients with COPD and suboptimal PIFR was not assessed. However, there were no new safety concerns, as very few AEs were reported for either treatment group; fewer AEs occurred with revefenacin than with tiotropium. Dyspnea and cough were the only treatment-related AEs reported in more than 2% of patients in either group. One SAE (a COPD exacerbation) was reported in the tiotropium group. AEs leading to permanent discontinuation of study drug were only reported in the tiotropium group (4.8%).^22^

Study 0167 (NCT03573817) was a randomized, double-blind, 2-period, parallel-group, 42-day phase 3b study that evaluated the safety and tolerability of revefenacin 175 µg when given either sequentially before or combined with formoterol 20 µg via a Pari LC Sprint jet nebulizer using the Pari Trek S compressor in patients with moderate to very severe COPD.^23^ The primary endpoint was the safety and tolerability of revefenacin when dosed sequentially with formoterol for 21 days. The secondary endpoint was the safety and tolerability of combined dosing as a mixture of revefenacin and formoterol for 21 days. Other LAMAs or LABAs were prohibited during the trial. At baseline, most patients were men (56%-58%) and/or current smokers (54%-59%), the mean age was 63 to 64 years, and the mean baseline postbronchodilator FEV_1_ was 55%.^23^

AEs were minimal across all groups, and there were no SAEs or clinically relevant changes in heart rate, QT interval corrected for heart rate using Fridericia’s method, vital signs, or laboratory results reported in any treatment group. The exploratory endpoint was the change in lung function from baseline on day 21 and day 42. A greater clinically relevant change from baseline in trough FEV_1_ was observed in the revefenacin and formoterol group vs the placebo and formoterol group during sequential (157.1 mL vs 53.3 mL) and combination (115.6 mL vs 35.0 mL) administration.^23^ Limitations of this study included its short length (42 days) and its lack of power to show differences between the treatments for the efficacy endpoints. Thus, further research and development into the long-term safety and efficacy of nebulized dual therapy will be required.^23^

## Place in therapy

Desirable characteristics for antimuscarinic agents used in the treatment of COPD include once-daily dosing, low rates of antimuscarinic AEs, and an effective, user-friendly delivery device. Clinical trial data on revefenacin demonstrated its clinical efficacy (in terms of improved FEV_1_) relative to both placebo and tiotropium among patients with moderate to very severe COPD.^14,17-23^ Overall, the data suggested that FEV_1_ was not significantly different between revefenacin and tiotropium.^21,22^ The clinical studies showed that revefenacin was well tolerated and was generally similar to tiotropium.^21,22^ In addition, revefenacin demonstrated a low incidence of antimuscarinic AEs, which is consistent with revefenacin’s pharmacological properties of competitive antagonism of the M_3_ receptor, unique molecular class (ie, the absence of a quaternary ammonia), and lung-selective design.^10,13^

Revefenacin may be a suitable alternative to inhalers in certain patient populations. A post hoc subgroup study of patients with markers of severe disease demonstrated that revefenacin via nebulization could benefit elderly patients, as well as those with cognitive or physical limitations.^24^ Additional treatment considerations include patient adherence. Revefenacin is the first nebulized LAMA administered once daily and offers an advantage over other twice-daily bronchodilators because it can potentially improve patient adherence. Dosing frequency has a major impact on medication adherence in patients with chronic diseases.^27^ Twice-daily dosing is frequency associated with a lower adherence rate than with once-daily dosing, with regimen adherence reduced by 13.1% and timing adherence reduced by 26.7%.^27^ Medication nonadherence can increase the risk for worsening COPD symptoms and COPD exacerbations.

In terms of delivery device, jet nebulizers, such as the Par LC Sprint, are easy to use and provide an efficient drug delivery system.^28^ In the 0167 study,^23^ revefenacin was administered via the same jet nebulizer with other nebulized bronchodilators (ie, formoterol), allowing for ease of administration and cleaning. However, further research and development is needed to evaluate the long-term safety, efficacy, and stability of nebulized dual therapy. It is important to consider infection control with nebulizers across all healthcare settings, given that bacteria grow in wet and moist environments. Nebulizers can be protected from contamination by following the manufacturers’ instructions for care and cleaning. However, additional factors should be taken into consideration given the current ongoing coronavirus disease 2019 (COVID-19) pandemic. Aerosol nebulization is considered to have a high risk of spreading COVID-19 to healthcare personnel. For inpatient use, guidance states to use personal protective equipment (including N95 masks and eyewear) and negative pressure rooms when possible.^29^ Additionally, placing a filter on the exhalation component of a nebulizer may provide protection against infection and minimize secondhand aerosol inhalation in hospitals and outpatient clinics.^30^ If these conditions cannot be met, then the use of inhalers may be preferred. Additionally, the American College of Asthma, Allergy, and Immunology released guidance for managing patients on nebulizers at home who have confirmed or suspected COVID-19. This guidance recommends using a nebulizer in an area where the air is not recirculated.^31^

In terms of cost, the wholesale acquisition cost for a monthly supply is $1,323.90 for revefenacin (Yupelri), which is similar to that for glycopyrrolate (Lonhala Magnair) at $1,359.60.^32^ This price is the most readily available reference price for clinicians; however, it does not provide a good estimate of the cost to patients (with the exception of patients who pay with cash). For patients with commercial insurance, the cost is mitigated by the use of manufacturer copayment cards, and patients often pay nothing for up to 12 months of therapy. Medicaid patients have little to no cost sharing. Medicare patients have standard payments based on the reimbursed amount under Medicare part D, normally paying 25% of the cost of the medication after their deductibles are met in addition to their monthly premium. This amount drops to 5% of the total cost of the drug once patients reach catastrophic coverage. Revefenacin has the advantage of being able to be billed under Medicare part B through a pharmacy or durable medical equipment supplier, unlike glycopyrrolate, which can only be billed under Medicare part D. This results in a 20% copayment for patients, which is mitigated by supplemental Medicare plans (F, N, etc) that reduce the copayment to $0 for patients.

## Conclusion

Revefenacin, a once-daily LAMA for use with a standard jet nebulizer, represents an important advance in the treatment of COPD. Revefenacin use has been shown to result in improvements in lung function and health status in patients with moderate to very severe COPD, including in patients with markers of more severe disease and patients who received concomitant LABA or LABA/ICS. Additionally, it was well tolerated, and AEs were generally mild without evidence of cardiovascular toxicity.

## Supplementary Material

zxab154_suppl_Supplementary_Appendix
