# Supplementary material for: Revefenacin, a once-daily, long-acting muscarinic antagonist, for nebulized maintenance therapy in patients with chronic obstructive pulmonary disease
Source: Am J Health Syst Pharm. 2021 Apr 4;78(13):1184–94. doi: 10.1093/ajhp/zxab154 (PMC8083528; doi:10.1093/ajhp/zxab154)
Supplement: zxab154_suppl_Supplementary_Appendix [file zxab154_suppl_supplementary_appendix.doc]

**eAppendix—Supplementary material**

**Data selection**

A PubMed search was conducted (January 2010 to September 2020) using the following terms/phrases: COPD, chronic obstructive, pulmonary disorder, LAMA, long-acting muscarinic antagonist, nebulized, revefenacin, standard jet nebulizer. Studies of revefenacin and articles relating to treatment in patients with COPD were identified.

**Dosage and administration**

Revefenacin is administered as a single inhalation of 175 µg once daily via a standard jet nebulizer connected to an air compressor. Revefenacin is supplied as 3 mL of revefenacin solution packaged in a unit-dose low-density polyethylene vial overwrapped in a foil pouch. Revefenacin does not require dilution prior to administration by nebulization, and the mean nebulization time is 8 minutes.^1^

**Drug interactions**

There are no human data available on specific drug-drug interactions. However, it is possible that revefenacin administration with other anticholinergic medications may increase the risk for anticholinergic AEs.^1^ Revefenacin administration with organic-anion-transporting polypeptide 1B1/1B3 inhibitors (for eg, rifampicin, cyclosporine) is not recommended as it may increase systemic exposure of THRX-195518.^1^

**Clinical trials**

**Phase 2 clinical trials - eligibility criteria**^2-4^. In addition to a diagnosis of stable, moderate to severe chronic obstructive pulmonary disease (COPD) (post-bronchodilator forced expiratory volume in 1 second [FEV_1_] greater than or equal to 30% and less than 80% of predicted normal values), the inclusion criteria for the clinical studies included patients aged ≥ 40 years and smoking history of at least 10 pack years. Typical exclusion criteria included significant respiratory disease other than COPD, respiratory tract infection or hospitalization within 12 weeks before study enrollment, and medical conditions that preclude the use of anticholinergic medications.

**Phase 3 clinical trials - eligibility criteria**^5-9^. In addition to a diagnosis of stable, moderate to very severe COPD (post-ipratropium FEV_1_ less than or equal to 80% of predicted normal values but at least 700 mL [at least 400 mL for study 0149]), the inclusion criteria for the clinical studies reviewed here included patients aged ≥40 years and smoking history of at least 10 pack-years. For study 0149, patients were required to have a peak inspiratory flow rate <60 L/min against the resistance of Diskus. Typical exclusion criteria included significant respiratory disease other than COPD, a history of myocardial infarction or unstable angina within the previous 6 months, respiratory tract infection or hospitalization within 6 or 8 weeks before study enrollment, and medical conditions that preclude the use of anticholinergic medications.

**Phase 2 and phase 3 clinical trials - definitions/clinical relevance.** The minimal clinically important difference (MCID) was used to determine clinical relevance. For trough FEV_1_, an increase ≥100 mL was considered clinically relevant.^10^ For St. George’s Respiratory Questionnaire a reduction in mean baseline score of 4 points was considered clinically relevant.^10^ There is no generally accepted MCID for the COPD Assessment Test , however a change of 2 points was clinically relevant and correlated well with the MCID of other validated health status measures.^11^ For Clinical COPD Questionnaire, the validated MCID is 0.4,^12^ and for Transition Dyspnea Index the difference between groups in total score ≥1 points.^10^

For safety, an adverse event (AE) was defined as any untoward medical occurrence associated with the use of the drug, whether or not considered drug-related. A serious AE was defined as occurrence at any dose that resulted in the following: death, life-threatening situation, hospitalization, persistent or significant disability/incapacity, congenital anomaly/birth defect, or requires intervention to prevent permanent impairment or damage. Major adverse cardiac events (MACEs) were determined through blinded adjudication. Adjudicated MACEs included death (all-cause, cardiovascular or non-cardiovascular), myocardial infarction/unstable angina, stroke/transient ischemic attack, heart failure, and cardiac arrhythmia (atrial and ventricular).

**Supplementary References**

1. US Food and Drug Administration. Highlights of prescribing information YUPELRI® (revefenacin) inhalation solution, for oral inhalation. 2018. https://www.accessdata.fda.gov/drugsatfda_docs/label/2019/210598s001lbl.pdf (accessed 2020 Jul 9).
2. Quinn D, Barnes C, Yates W, et al. Pharmacodynamics, pharmacokinetics and safety of revefenacin (TD-4208), a long-acting muscarinic antagonist, in patients with chronic obstructive pulmonary disease (COPD): results of two randomized, double-blind, phase 2 studies. *Pulm Pharmacol Ther*. 2018;48:71-79. doi: 10.1016/j.pupt.2017.10.003
3. US National Library of Medicine: Clinicaltrials.gov. A 7-day cross-over study of QD (once daily) and BID (twice daily) TD-4208 in chronic obstructive pulmonary disease(COPD). Available from:https://www.clinicaltrials.gov/ct2/show/study/NCT02109172?term=Revefenacin&rank=9 (accessed 2020 September 28).
4. Pudi K, Barnes C, Moran E, et al. A 28-day, randomized, double-blind, placebo-controlled, parallel group study of nebulized revefenacin in patients with chronic obstructive pulmonary disease. *Respir Res*. 2017;18:182. doi: 10.1186/s12931-017-0647-1
5. Ferguson G, Feldman G, Pudi K, et al. Improvements in lung function with nebulized revefenacin in the treatment of patients with moderate to very severe COPD: results from two replicate phase III clinical trials. *Chronic Obstr Pulm Dis*. 2019;6:154-65. doi: 10.15326/jcopdf.6.2.2018.0152
6. Donohue J, Kerwin E, Sethi S, et al. Revefenacin, a once-daily, lung-selective, long-acting muscarinic antagonist for nebulized therapy: safety and tolerability results of a 52-week phase 3 trial in moderate to very severe chronic obstructive pulmonary disease. *Respir Med*. 2019;153:38-43. doi: 10.1016/j.rmed.2019.05.010
7. Donohue J, Kerwin E, Sethi S, et al. Maintained therapeutic effect of revefenacin over 52 weeks in moderate to very severe chronic obstructive pulmonary disease (COPD). *Respir Res*. 2019;20:241. doi: 10.1186/s12931-019-1187-7
8. Mahler D, Ohar J, Barnes C, et al. Nebulized versus dry powder long-acting muscarinic antagonist bronchodilators in patients with COPD and suboptimal peak inspiratory flow rate. *Chronic Obstr Pulm Dis*. 2019;6:321-331. http://doi.org/10.15326/jcopdf.6.4.2019.0137
9. Siler T, Moran E, Yun J, et al. Safety and efficacy of revefenacin and formoterol in sequence and combination via a standard jet nebulizer in patients with chronic obstructive pulmonary disease: a phase 3b, randomized, 42-day study. *Chronic Obstr Pulm Dis*. 2020;7:99-106. <http://doi.org/10.15326/jcopdf.7.2.2019.0154>
10. Jones PW, Beeh KM, Chapman KR, et al. Minimal clinically important differences in pharmacological trials. *Am J Respir Crit Care Med*. 2014;189:250-5. doi:10.1164/rccm.201310-1863PP
11. Kon SS, Canavan JL, Jones SE, et al. Minimum clinically important difference for the COPD Assessment Test: a prospective analysis. *Lancet Respir Med.* 2014;2(3):195-203. doi: 10.1016/S2213-2600(14)70001-3
12. Kocks JW, Tuinenga MG, et al. Health status measurement in COPD: the minimal clinically important difference of the clinical COPD questionnaire. *Respir Res*. 2006;7(1):62**.** doi: 10.1186/1465-9921-7-62
